# AutoSyP: A Low-Cost, Low-Power Syringe Pump for Use in Low-Resource Settings

**DOI:** 10.4269/ajtmh.16-0285

**Published:** 2016-10-05

**Authors:** Alexa Juarez, Kelley Maynard, Erica Skerrett, Elizabeth Molyneux, Rebecca Richards-Kortum, Queen Dube, Z. Maria Oden

**Affiliations:** 1 Rice 360: Institute for Global Health Technologies, Rice University, Houston, Texas; 2 Basic Health International, San Salvador, El Salvador; 3 Department of Bioengineering, Rice University, Houston, Texas; 4 Department of Paediatrics, Queen Elizabeth Central Hospital, Blantyre, Malawi

## Abstract

This article describes the design and evaluation of AutoSyP, a low-cost, low-power syringe pump intended to deliver intravenous (IV) infusions in low-resource hospitals. A constant-force spring within the device provides mechanical energy to depress the syringe plunger. As a result, the device can run on rechargeable battery power for 66 hours, a critical feature for low-resource settings where the power grid may be unreliable. The device is designed to be used with 5- to 60-mL syringes and can deliver fluids at flow rates ranging from 3 to 60 mL/hour. The cost of goods to build one AutoSyP device is approximately $500. AutoSyP was tested in a laboratory setting and in a pilot clinical study. Laboratory accuracy was within 4% of the programmed flow rate. The device was used to deliver fluid to 10 healthy adult volunteers and 30 infants requiring IV fluid therapy at Queen Elizabeth Central Hospital in Blantyre, Malawi. The device delivered fluid with an average mean flow rate error of −2.3% ± 1.9% for flow rates ranging from 3 to 60 mL/hour. AutoSyP has the potential to improve the accuracy and safety of IV fluid delivery in low-resource settings.

## Introduction

An estimated 15 million premature babies are born every year, and preterm birth is responsible for more than 1 million neonatal deaths.^1^,^2^ Preterm infants are at increased risk of dying from various complications, including hypoglycemia, electrolyte imbalance, and dehydration, for which proper treatment often requires a syringe pump to accurately control intravenous (IV) fluid administration.^3^ Born Too Soon: The Global Action Report on Preterm Birth identified syringe pumps as one of the nine most pressing technical innovations needed to improve care for preterm babies.^2^ Similarly, the World Health Organization (WHO) identified syringe pumps that deliver IV medications as an essential medical device that should be present at all district hospitals for care of mothers, newborns, and children.^4^ Because of their high cost and complexity, commercial-grade syringe pumps are often not readily available in low-resource settings, where the majority of preterm deaths occur.^5^,^6^

Without syringe or infusion pumps, hospitals in low-resource settings deliver fluids with gravity-driven IV drip systems.^7^ Small neonates require IV fluid to be dispensed at rates ranging from 60 to 100 mL/kg/day.^8^ For example, a 1.5 kg baby receiving 150 mL of fluids per day requires a flow rate of approximately 6 mL/hour, which is challenging to control with an IV drip system. These systems have a higher risk of over- or underhydration due to imprecise flow rate control and lack of monitoring.^9^

One alternative method for fluid delivery is Springfusor (Go Medical Industries, Subiaco, Western Australia), a mechanically driven syringe pump. The Springfusor syringe driver is available for 10- and 30-mL syringe sizes and can be used repeatedly on different patients. However, Springfusor has several limitations that present barriers to adoption in low-resource settings. Although the syringe driver is reusable and costs $45 for the 10-mL syringe driver and $52 for the 30-mL syringe driver, each infusion requires a disposable flow control tube, which costs an additional $4.40–$9.40.^10^ Precise control of flow rate also requires that the user calculates a correction factor depending on the fluid delivered and the environmental conditions, which can be time consuming in an already time-constrained environment. In addition, Springfusor can only be used with Braun syringes, creating a challenge for hospitals where the supply chain for disposables can be unreliable.^11^

Thus, there is an important global need for affordable, accurate, and easy-to-use tools to enable safe delivery of IV fluids to newborns and infants in low-resource settings. To meet this need, we designed AutoSyP, an automatic syringe pump for controlled delivery of IV fluids in low-resource settings. This article describes the design of the AutoSyP device as well as pilot clinical trials to evaluate its performance in healthy adult volunteers and in infants requiring IV fluids in a low-resource hospital.

## Materials and Methods

### Device design.

**Figure 1.**
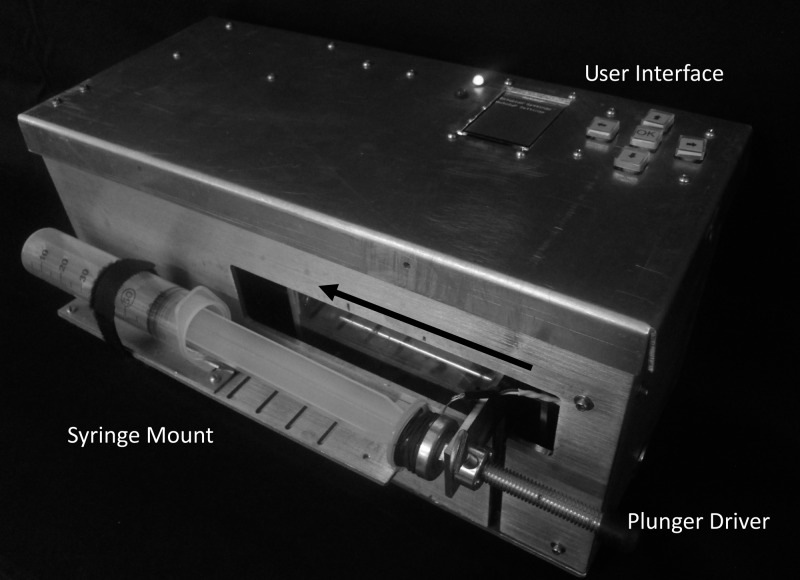
AutoSyP clinical evaluation system.

The AutoSyP device is designed to meet the International Electrotechnical Commission (IEC) standards for accuracy, and to be low cost, robust, and low power. The user interface is designed to be simpler than those of commercially available syringe pumps to decrease the risk of user error. The current prototype can fit syringe sizes of 5, 10, 20, 30, and 60 mL and run from 3 to 60 mL/hour (Figure 1

). A constant-force spring within the device provides energy to depress the syringe plunger, replacing a significant portion of the electrical energy that is normally used in syringe pumps. The spring is set manually by the user between uses by pulling the depressor to the right edge of the device. The rate of energy release from the spring, and thus the infusion flow rate, is controlled by a microcontroller and a stepper motor within the device.

Figure 2

**Figure 2.**
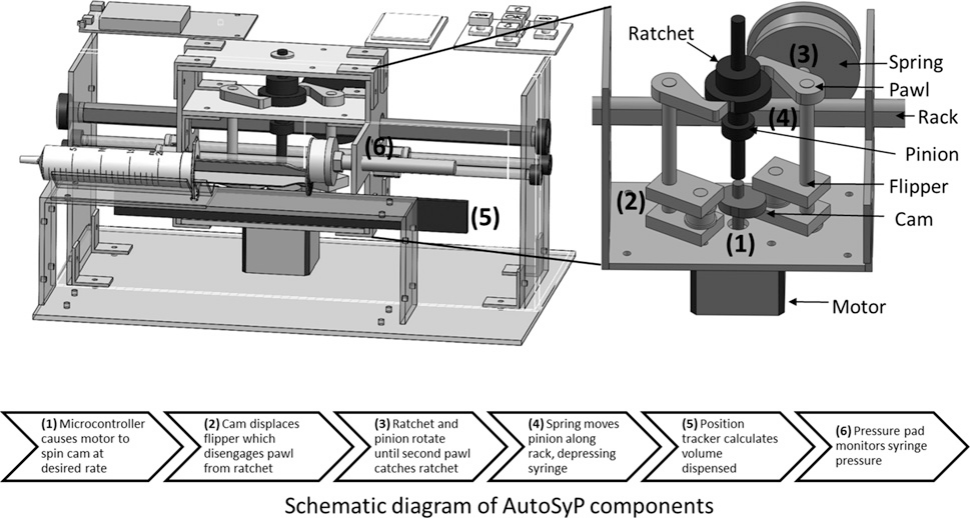
The spring-gear translation mechanism transfers the spring's potential energy to the syringe plunger to deliver fluid.

shows a schematic diagram of AutoSyP. The device incorporates a rack and pinion system that uses energy stored in the spring to depress the syringe plunger. The syringe plunger is attached to the rack, and the spring is used to move the pinion along the rack at a rate dictated by a microcontroller. The microcontroller (Figure 2) causes a motor to spin a cam shaft at the programmed rate. The cam displaces a flipper, which disengages the pawl from the ratchet. The disengaged pawl allows the ratchet and pinion to freely rotate. This allows the spring to move the pinion along the rack and depress the syringe plunger. Movement stops when the second pawl catches the ratchet and locks the pinion in position. The inclusion of two pawls with the ratchet ensures that each motor rotation results in one pinion rotation and thus, one total advancement step. Even though the advancement of the plunger is triggered by rotation of the motor, it is driven by the potential energy stored in the spring.

A volume tracker uses a membrane potentiometer to monitor the movement of the depressor. The change in distance directly correlates to the movement of the syringe plunger and thus the amount of fluid dispensed. This amount is displayed on the user interface and is used to monitor the accuracy of the infusion. If the calculated flow rate deviates by more than 10% from the programmed flow rate, an accuracy alarm is triggered and the infusion stops. A pressure sensor monitors the pressure between the plunger driver and the syringe plunger, as a surrogate for the pressure in the IV line. If the pressure exceeds a preprogrammed threshold during the infusion, the device stops and the alarm system is triggered. This notifies the nurse to check the infusion site for signs of an occlusion. The occlusion alarm threshold was set to alarm at a pressure that is 50 mmHg higher than the pressure measured during the initial 10 motor steps of the infusion for pediatric patients and 100 mmHg higher for adults.

### Device use.

AutoSyP is operated in a similar manner to commercial-grade syringe pumps. First, the user pulls back the device plunger to unwind the spring. The syringe and extension set are prepared with the prescribed fluid and the syringe is secured to the device. The user enters the syringe brand, syringe size, and infusion rate using the five buttons and screen on the user interface. The user interface displays the volume dispensed, time remaining, and set infusion rate during the infusion. During the infusion, the user has the option to pause the infusion, adjust the initial infusion settings, or stop the infusion. Visual and audio alarms programmed on the device indicate pressure occlusions, low accuracy, empty syringe, or low battery level.

### Calibration and laboratory testing protocols.

Calibration was performed using syringe sizes of 10, 20, 30, and 60 mL and intended flow rates of 5, 10, and 60 mL/hour. The syringe was prepared with an IV line and catheter for each calibration test to mimic clinical use. The IV line was primed, and the syringe was filled to its maximum volume before being loaded on the AutoSyP device. The rate was set on the user interface, and the device was programmed to dispense all fluid in the syringe. During the infusion, a scale connected to a data recording system tracked the mass of fluid dispensed into a cup at the distal end of the IV catheter. The mass was multiplied by the density of the water to convert it to volume. Using a serial monitor connected to AutoSyP's microcontroller, the time and number of half rotations (“steps”) completed by the device was continuously recorded. Each step from the serial port was time matched with the fluid dispensed from the scale data. The amount of volume dispensed at each step was averaged to determine the average volume dispensed per step. The calibration protocol was completed three times for each syringe size and rate. The values from the three calibration tests were averaged to determine the final calibration number (mL/step). For all tests, the data from the linear potentiometer were collected with the serial monitor to determine the correlation between the linear potentiometer value and volume dispensed at each step. This correlation was used to calibrate the device's estimation of current volume dispensed based on the linear potentiometer value.

Following calibration, benchtop accuracy testing was performed with 10-, 20-, 30-, and 60-mL syringe sizes. Each syringe was filled to its maximum volume and separate tests were conducted at 3, 5, 10, 40, and 60 mL/hour flow rates at back pressures of 0, 50, and 100 mmHg to mimic clinical back pressures.^12^ The entire fluid volume was dispensed and data from the scale recording system were used to measure the amount of fluid dispensed over the programmed protocol time. Back pressure was generated by placing the distal end of the IV catheter at a specified vertical displacement above the syringe pump and placing it in water.

Data from each test were collected in accordance to the IEC standards for electrically driven portable infusion devices (IEC-60601).^13^ IEC standards do not have set standards to calculate mean flow rate over a complete infusion for infusion periods shorter than 2 hours or flow rates greater than 5 mL/hour. Therefore, the mean flow rate equation for nonelectrically driven portable infusion devices (International Organization for Standardization [ISO]-286020) was used to calculate the average flow rate for each infusion.^14^ The mean flow rate, *Q*_m_, was determined by measuring the time, *T*, necessary for the device to deliver 75% of the nominal volume, *V*_n_, of the solution. This was calculated using the equation *Q*_m_ = (0.75 × *V*_n_)/*T* (ISO-286020). This method allows for flow rate accuracy comparisons to be made for all infusion periods and flow rates.

Trumpet curves were generated following the IEC 60601 standard to assess the variations in flow accuracy throughout the infusion. Data were analyzed to determine the accuracy of fluid delivery over specific observation windows across the second to sixth hour of a 5 mL/hour infusion. Observation windows were aligned with the time it took AutoSyP to complete 1, 2, 5, 11, 19, and 31 motor steps. The minimum and maximum flow rate percent errors were calculated for each window.

In accordance with IEC 60601 standards, the maximum time for activation of the occlusion alarm and the unintended bolus volume were determined with 5-, 20-, and 60-mL syringes set to dispense at 5 mL/hour, the required flow rate for IEC occlusion testing.

Battery life was tested using a new, fully charged cell. The device was operated continuously at a rate of 5 mL/hour until the battery voltage dropped below 7 V, the minimum voltage recommended by the manufacturer for powering the microcontroller.

Percent errors were calculated for the mean flow rate error. The calculated mean flow rate was compared with the programmed flow rate to calculate the mean flow rate error. Percent errors were compared with the errors reported in manufacturers specifications for commercial syringe pumps (±5%) since the IEC standards do not set a percent error limit or target.

### Clinical evaluation protocol.

Evaluation of the clinical accuracy and usability of the device was carried out in a 2-phase clinical trial at Queen Elizabeth Central Hospital (QECH) in Blantyre, Malawi. The study protocol was approved by the National Health Science Research Committee of the Malawi Ministry of Health (protocol no. 1304) and the institutional review board at Rice University (protocol no. 14-150F). In the first phase, 10 trials were performed. Infusions of normal saline were administered to healthy adults at flow rates ranging from 3 to 60 mL/hour with a 10-, 20-, or 60-mL syringe. This initial study aimed to mimic pediatric infusions and validate the safety and accuracy of the device in a healthy, low-risk population.

In the second phase of the study, the accuracy and usability of AutoSyP was evaluated in its intended clinical setting with a target patient population. In this phase, 30 babies in the pediatric department at QECH who had been prescribed IV fluid infusions were enrolled in the study. Programmed flow rates and syringe sizes were based on clinician indication according to the specific needs of the patient. Three staff nurses were trained on AutoSyP use, clinical preparations, and study protocol and recruitment procedures. These nurses managed patient recruitment and consent, programmed infusion settings on the device, and prepared the IV lines and cannulas during the study. During the infusion, a nurse closely monitored the device and baby every hour for any infusion-related complications. In addition, trained technicians monitored the technical performance of the AutoSyP device every 30 minutes and intervened if there were any technical complications. If the device malfunctioned or an alarm was triggered, the technician recorded the event and solution on the patient's monitoring form to track device performance. If the subject's guardian, the nurse, or research technician indicated concern during the infusion, the treatment was discontinued and the subject received treatment as necessary using the standard IV drip system or a commercial syringe pump if it was available.

Data were logged using the serial monitor to track fluid dispensed, number of motor steps, and any triggered alarms. Percent errors were calculated for mean flow rate error. The mean flow rate equation for nonelectrically driven portable infusion devices (ISO-286020) and the logged data were used to calculate the average flow rate for each clinical infusion. The calculated mean flow rate was compared with the programmed flow rate to calculate the mean flow rate error.

## Results

### Benchtop testing.

Figure 3

**Figure 3.**
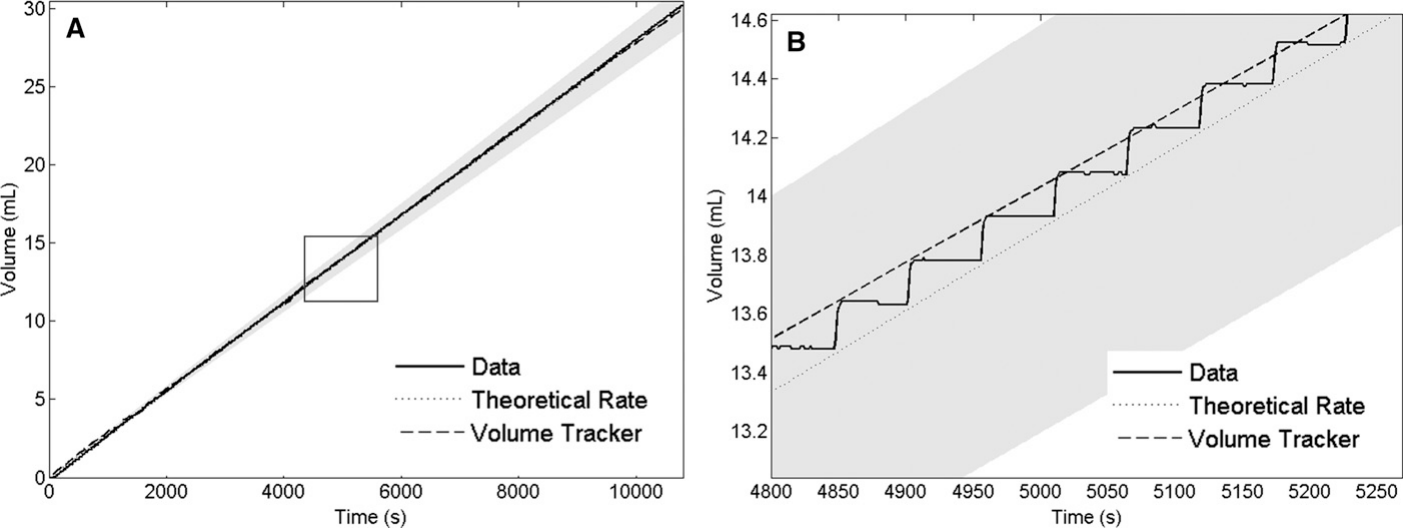
(**A**) Example benchtop accuracy test data for a 30-mL syringe at 10 mL/hour. (**B**) Magnified benchtop accuracy data for the same infusion. The solid line represents volume delivered based on the mass data and the dashed line represents the volume delivered based on AutoSyP's internal volume delivery tracking mechanism. The dotted line represents the theoretical volume and the gray area is the ±5% error range.

shows an example of the volume dispensed (solid line) over time compared with the volume tracker (dashed line), which measured the position of the syringe plunger and the intended fluid delivery rate (dotted line) for a 30-mL infusion at a rate of 10 mL/hour. As shown in Figure 3B, a small volume is dispensed with each motor step. For rates ranging from 3 to 60 mL/hour, the device had a mean flow rate error of 1.76% ± 1.41% in laboratory conditions (Table 1). Changing the back pressure from 0 to 100 mmHg did not affect the mean flow rate error (*P* = 0.47).

Figure 4

**Figure 4.**
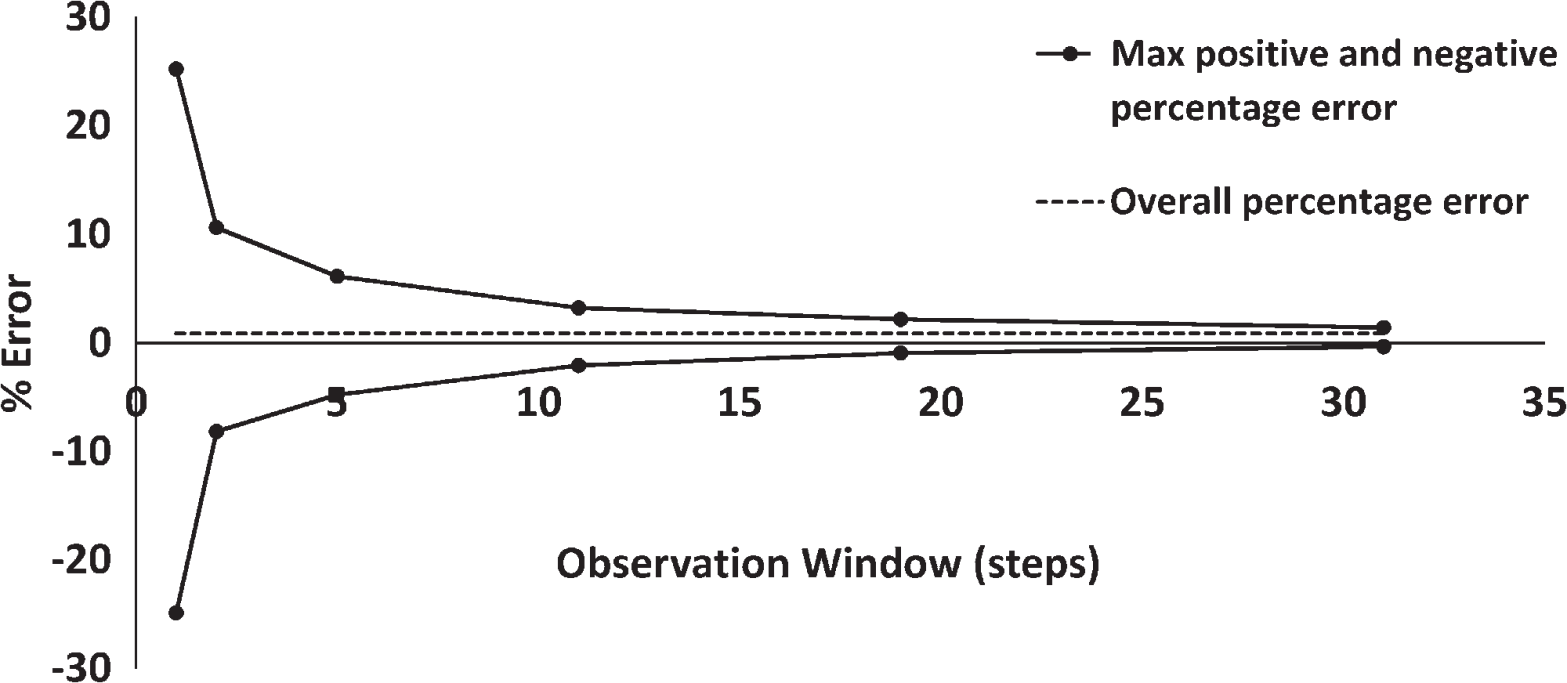
Accuracy of fluid delivery as function of observation windows at 1, 2, 5, 11, 19, and 31 steps were measured to produce the trumpet curve for a 60-mL syringe at 5 mL/hours. The dashed line represents the overall percentage error of the infusion. The solid black line represents the overall percentage error of the second hour of the infusion.

shows an example trumpet curve. As expected, the flow rate accuracy improves as fractional errors from larger observation windows are averaged. The flow rate error is approximately 25% at the smallest observation window, but decreases to less than 1% at the largest observation window.

**Figure 5.**
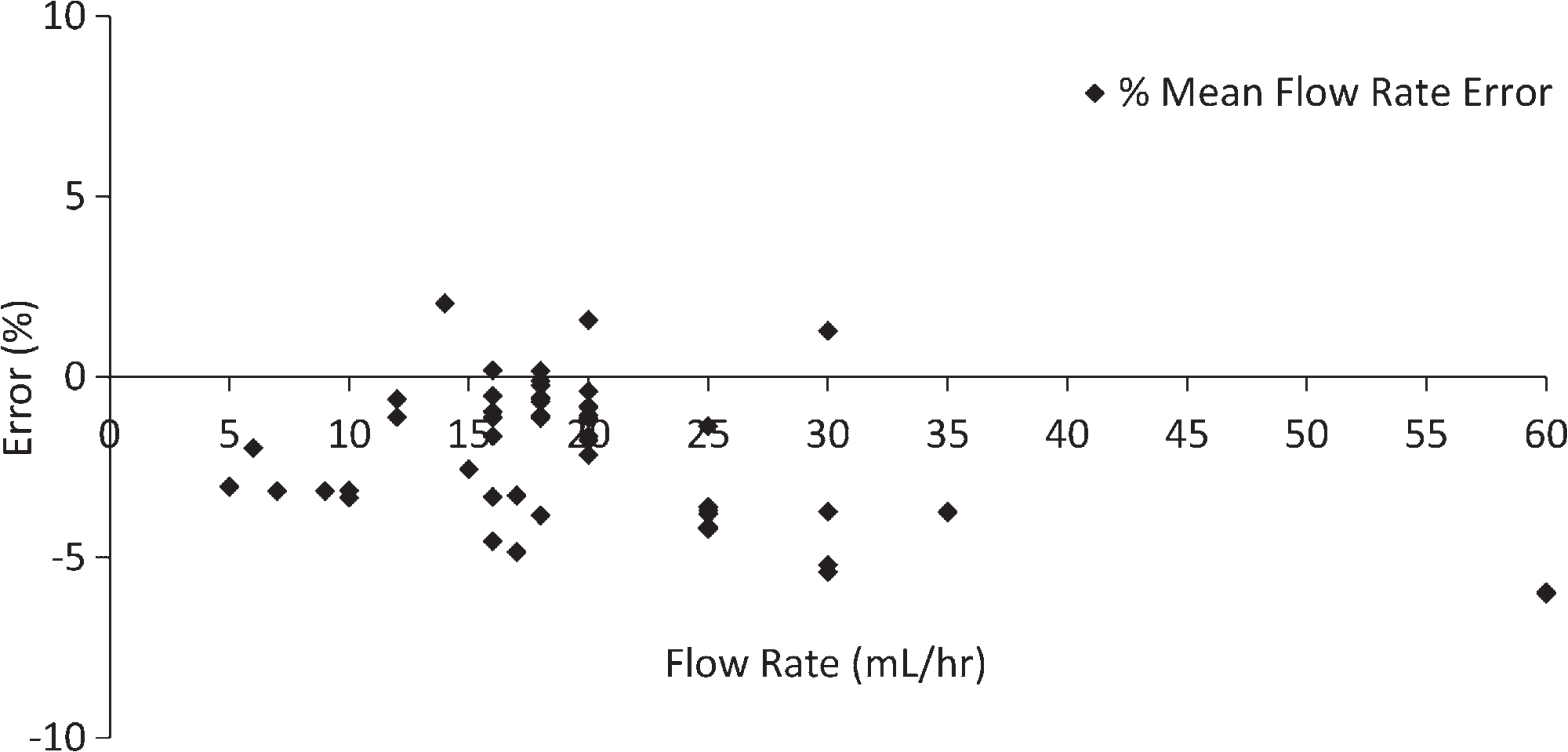
Mean flow rate error as a function of flow rate for each infusion. Grey area represents ±5% error region for mean flow rate.

The average time to alarm and bolus volumes were 1.4, 4.15, and 8.64 minutes and 0.25, 0.30, and 0.66 mL for the 5-, 20-, and 60-mL syringes, respectively. On average, the occlusion was triggered within three motor steps for all syringe sizes. Battery test results showed that AutoSyP can run continuously on battery power for up to 66 hours.

### Clinical performance.

In the first phase of clinical testing, there was less than 3% error in the mean flow rate for all 10 healthy volunteer trials (Table 2). A single infusion was defined as the complete infusion of one syringe. In trials three and four, patients received multiple infusions using the same syringe size and flow rate. The average mean flow rate error over the 10 trials was −0.60% ± 1.65% (95% confidence interval [CI]: −1.61, 0.42). No true occlusions or device occlusion alarms occurred. There were also no malfunctions of the device.

In the second phase, 30 pediatric patients were enrolled from the Chathinka Neonatal Intensive Care Ward, the Pediatric Nursery High Dependency Unit, and the Pediatric Special Care Ward. About 59 infusions were completed in this phase. Patients were given one or multiple infusions of normal saline, dextrose, Ringer's lactate (with or without added potassium chloride) for treatment of dehydration, electrolyte imbalance, and/or hypoglycemia based on physicians' orders (Table 3). The age of subjects ranged from 2 days to 9 months and weights ranged from 1.2 to 7.8 kg.

Prescribed flow rates varied between 5 and 60 mL/hour, and prescribed volume delivered per infusion varied between 8 and 60 mL. Syringe sizes for all infusions were 60 mL. The average for mean flow rate error over the 59 infusions was −2.27% ± 1.91% (95% CI: −2.76, −1.78) (Figure 5).

During the pediatric patient study, there were 26 infusions where the occlusion alarm was triggered one or multiple times. The occlusion alarm alerted the nurse a total of 59 times, and 17 of the triggered occlusion alarms were false alarms. About 42 alarms were true positives, where the IV line occluded and stopped fluid delivery temporarily until a nurse could correct the occlusion. Two patients were discharged early and treatment with AutoSyP was discontinued.

## Discussion

AutoSyP is a low-power, low-cost device that meets IEC standards for flow rate accuracy, occlusion alarm testing, and trumpet curves for electrical syringe pumps and has the potential to be useful in pediatric wards in low-resource settings. Our pilot study showed that AutoSyP delivered fluid with a mean flow rate error of 1.76% in benchtop tests at rates ranging from 3 to 60 mL/hour. The 10 infusions completed in healthy adult volunteers were delivered with a −0.6% mean flow rate error. In 59 infusions completed in pediatric patients, the mean flow rate error was −2.27%. The mean flow rate error of AutoSyP is comparable to most electronic commercial-grade syringe pumps, in which flow rate error ranges from ±2% to ±5%.^15^,^16^ AutoSyP's battery operating time is 66 hours at 5mL/hour with a rechargeable battery, which is 11 times longer than that of the Alaris (Smith Medical ASD, Inc., Dublin, OH) and Medfusion (Becton, Dickinson and Company, Franklin Lakes, NJ) syringe pumps.^15^,^16^

The major strengths of the clinical study included testing in the intended setting and with the intended end users. The clinical trials took place in the pediatric wards at QECH and nurses from the wards were hired to set up the device and IV fluids for each patient. AutoSyP was exposed to the environmental challenges in the intended setting (i.e., power outages, line voltage spikes, high temperature, and humidity) and maintained functionality and accuracy. Feedback collected from pediatric nurses and physicians assessed the usability of the device in the wards and to determine design changes needed. Strengths of the device noted by users included the simplified and durable design of the device and the rechargeable battery power source. Users stated that it was easier to program infusions using AutoSyP than standard commercial pumps. The staff also noted that the long battery life of the device decreased the risk of the infusion interruptions due to power outages.

The limitations of the study included the continuous presence of technical support in the wards during infusions with AutoSyP, which would not be representative of permanent, long-term use of the device. Technical support was included to mitigate risks during the pilot study and provide immediate troubleshooting if a malfunction occurred. Future studies should minimize technical support when the device is regularly being used in the intended setting. Device limitations of AutoSyP included the large size of the device and low specificity of the occlusion alarm. AutoSyP is bigger and bulkier than current commercial syringe pumps. Although this did not hinder the performance, the device could not easily fit near the patient without adding benches to accommodate the device. The size was not practical for the pediatric ward because most available space was used for patients and their caregivers. The occlusion alarm had 17 false occlusion alarms when testing in pediatric patients. A majority of these alarms were triggered due to patient movements that caused a brief increase in pressure. In future design iterations, decreasing the device size and refining the occlusion alarm while maintaining accuracy will be a key element in the design criteria.

Premature babies in low-resource hospitals require precise IV administration of fluids to treat high-risk illnesses.^2^ The WHO recommends district hospitals have at least one syringe pump for every two beds located in the pediatric ward to provide controlled fluid delivery to patients.^4^ However, the majority of electric pumps are expensive, complex to use, and often break down, leaving this population at a high risk for under- or overdosage when receiving fluids via the alternative gravity-driven IV drip systems.^3^,^6^ Most commercially available syringe pumps cannot meet the needs of district hospitals in this setting, limiting the ability to meet the WHO guidelines. In light of the needs of low-resource hospitals, AutoSyP can provide a low-cost and durable option to district hospitals while also maintaining the high accuracy level seen in commercial-grade syringe pumps. This device will enable nurses and clinicians to provide controlled fluid delivery to severely ill patients and improve care for babies, pregnant women, and adults in the low-resource settings.

## Figures and Tables

**Table 1:** Benchtop accuracy test results

Trial no.	Flow rate (mL/hour)	Syringe size (mL)	Total volume delivered (mL)	Back pressure (mm Hg)	Mean flow rate error (%)
1	60	60	60	0	2.91
2	40	60	60	0	2.54
3	5	60	60	0	1.52
4	10	30	30	0	2.69
5	5	20	20	0	1.56
6	3	10	10	0	0.75
7	60	60	60	50	2.45
8	60	60	60	100	3.35
9	40	60	60	100	3.50
10	5	60	60	100	1.78
11	10	30	30	100	0.89
12	5	20	20	100	3.69
13	3	10	10	100	1.49

**Table 2:** Healthy volunteer study results

Trial no.	Flow rate (mL/hour)	Syringe size (mL)	Total volume delivered (mL)	No. of infusions	Mean flow rate error (%)
1	20	20	20	1	−0.41
2	3	10	9	1	1.05
3	15	60	120	2	−2.65
4	15	60	120	2	−2.90
5	5	10	10	1	−1.04
6	10	60	60	1	−1.30
7	20	20	20	1	−1.21
8	5	10	10	1	0.71
9	10	60	55	1	−0.80
10	3	10	10	1	2.51

**Table 3:** Pediatric study demographics

Total patients	30
Sex	
Male	10
Female	20
Age	
< 1 month	7
1–3 months	2
3–6 months	14
> 6 months	5
Not reported	2
Weight	
< 3 kg	5
3–6 kg	17
6–10 kg	6
> 10 kg	1
Not reported	1
Fluids delivered	
Normal saline + 10% dextrose	4
Normal saline + KCl	1
Ringer's lactate	3
Ringer's lactate + KCl	1
Ringer's lactate + 5–10% dextrose	19
Ringer's lactate + 5–10% dextrose + KCl	2
Indications for use	
Rehydration/maintenance fluid/fluid replacement	27
Electrolyte imbalance	2
Hypoglycemia	1
